# The Beat

**Published:** 2009-09

**Authors:** Erin E. Dooley

## Short Nights, Long-Term Health Effect?

In a study published online 30 June 2009 ahead of print in *The Journal of Clinical Endocrinology and Metabolism*, Arlet Nedeltcheva et al. report that repeatedly getting fewer than 6 hr of sleep per night could contribute to factors that can increase the long-term risk of type 2 diabetes. Participants were allowed to eat freely but slept either 5.5 or 8.5 hr each night, with the shorter duration associated with reduced oral glucose tolerance and insulin sensitivity and increased glucose effectiveness. Environmental factors such as noise and light pollution can lead to sleep deprivation.

## E-Cigarettes: Not Quite Healthy

**Figure:**
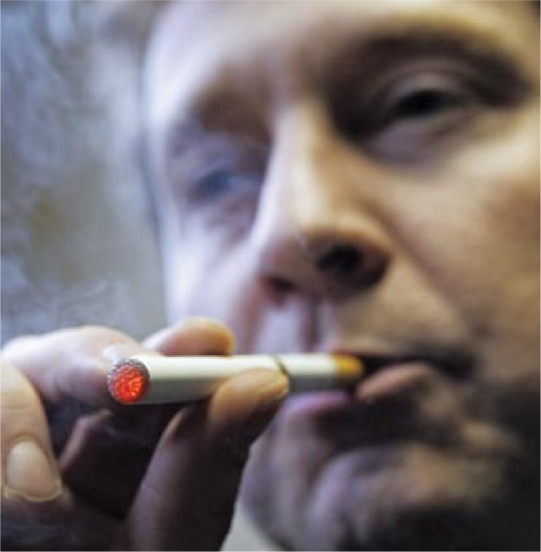
Water vapor “smoke” and a glowing LED tip make e-cigarettes look like the real thing.

In July 2009 the FDA released its analysis of 19 varieties of tobacco-less “electronic cigarettes.” Produced mainly in China, e-cigarettes are battery-charged devices that heat a nicotine/propylene glycol solution, producing a mist the smoker inhales. Although smokeless e-cigarettes are touted as safer than traditional cigarettes, the FDA found they still deliver detectable levels of known carcinogens and varying levels of nicotine, with 1 e-cigarette delivering twice the nicotine approved by the FDA for smoking cessation aids. Israel, Australia, Canada, and Mexico have banned e-cigarettes.

## A More Granular Look at Food Deserts

Areas where residents have limited access to affordable nutritious food—known as “food deserts”—have been named as a possible factor in the rise in U.S. obesity rates. But in *Access to Affordable and Nutritious Food*, a June 2009 report to Congress, the USDA reports that lack of access to nutritious foods may be a less important factor in obesity than relatively easy access to all other foods. “Many of the stores that carry [fruits and vegetables, whole grains, and low-fat milk] at low prices also carry all the less healthy foods and beverages as well,” write the authors. “Without also changing the dietary behaviors of consumers, interventions aimed at increasing access to healthy foods may not be successful in addressing obesity.”

## World Fisheries Still Afloat

**Figure:**
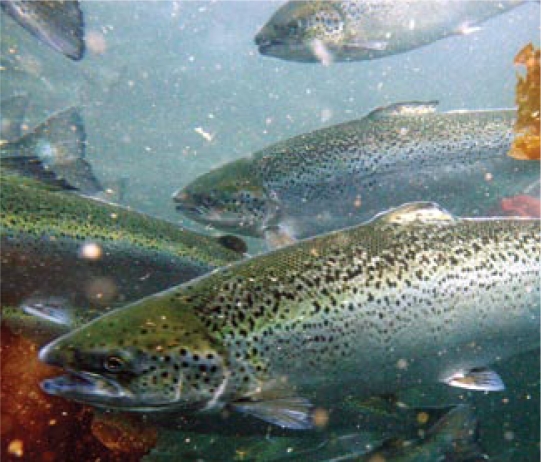
Nearly two-thirds of fisheries are in trouble, but recovery is possible.

Boris Worm et al. report in the 31 July 2009 issue of *Science* that even though many wild fish populations worldwide are close to collapse—63% of stocks assessed need rebuilding—careful management is beginning to pay off. Half of the 10 regions assessed, including the United States, Iceland, and New Zealand, have cut exploitation rates (the proportion of a total population of fish that are caught), which is a primary factor leading to collapse. The authors add that a global effort is needed to protect against further depletion and collapse, including measures that address the special needs of developing nations, where “most fishers do not have access to alternative sources of food, income, and employment,” the authors write.

## Government to Rein In Agricultural Antibiotic Use

In July 2009 the FDA announced its intentions to ban antibiotic use for promoting growth in farm animals and require veterinarian supervision of other agricultural uses of these drugs in order to reduce antibiotic resistance in humans. Two million Americans acquire bacterial infections during hospital stays every year, with 70% of the infections resistant to at least 1 antibiotic. Earlier, the House had introduced phaseout bills banning 7 classes of antibiotics from agricultural use and restricting antibiotics to therapeutic and preventive uses. FDA Deputy Commissioner Joshua Sharfstein testified before the House Committee on Rules that such restrictions will not compromise food safety.

## Styrene Reprieve in California

On 12 August 2009, California Superior Court judge Shelleyanne Chang preliminarily ruled that styrene monomer, a chemical used in the manufacture of items including food packaging and plastics, can be exempted from listing under the state’s Proposition 65 rule. Prop 65 requires that businesses post warnings about products containing known and possible carcinogens. In her ruling, Chang said styrene, which is classified as “possibly carcinogenic” by IARC, is crucial to the transport and sale of the state’s $1.6 billion berry crop and that a Prop 65 listing “could have a devastating effect on that product’s use.” Chang has 90 days to issue a final ruling on the matter.

**Figure:**
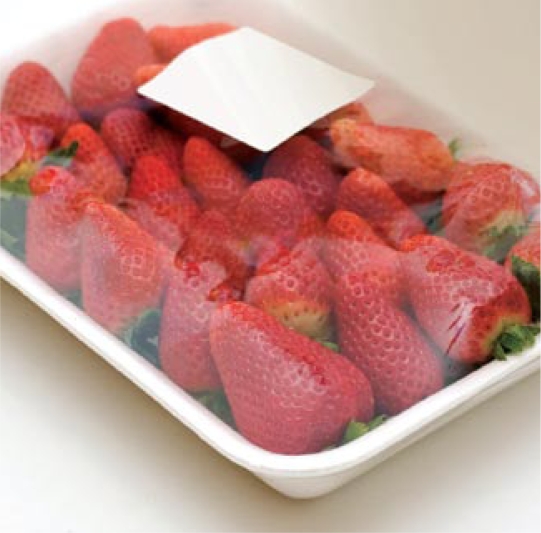
A California judge ruled that styrene is crucial to packaging the state’s strawberry, blueberry, and raspberry crops.

